# Placental Histopathology and Adverse Perinatal Outcomes in ART Pregnancies: A Comparative Analysis of Homologous and Heterologous Techniques

**DOI:** 10.3390/diagnostics16142192

**Published:** 2026-07-14

**Authors:** Eleonora Nardi, Simone Grassi, Andrea Costantino, Gian Franco Zannoni, Antonio Oliva, Vincenzo Arena

**Affiliations:** 1 Section of Anatomic Pathology, Department of Laboratory and Hematological Sciences, Fondazione Policlinico Universitario A. Gemelli IRCCS, Department of Life Sciences and Public Health, Università Cattolica del Sacro Cuore, 00168 Rome, Italy; gianfranco.zannoni@policlinicogemelli.it (G.F.Z.); vincenzo.arena@unicatt.it (V.A.); 2 Department of Health Sciences, Section of Forensic Medical Sciences, University of Florence, 50134 Florence, Italy; simone.grassi@unifi.it (S.G.); andrea.costantino1@unifi.it (A.C.); 3 Institute of Public Health, Section Legal Medicine, Catholic University, 00168 Rome, Italy; antonio.oliva@unicatt.it

**Keywords:** infertility, assisted reproductive technology, pregnancy, placenta, histopathology

## Abstract

**Background**: Assisted Reproductive Technology (ART) is associated with increased maternal and fetal complications. This study compared adverse outcomes and placental histopathology findings between ART and spontaneous conceptions, with a novel focus on homologous versus heterologous techniques. **Methods**: This retrospective cohort study included 80 singleton ART pregnancies (45 homologous, 35 heterologous) and 80 matched spontaneous controls delivered at A. Gemelli Hospital. Clinical data and placental lesions, classified via the Amsterdam criteria and Benton framework, were statistically analyzed. **Results:** ART pregnancies showed higher rates of preterm birth (mean Gestational Age: 37.97 vs. 38.72 weeks), NICU admissions (15% vs. 0%), and maternal vascular malperfusion (MVM). Heterologous ART was associated with significantly higher maternal age (42.6 vs. 37.8 years; *p* = 0.007) and lower gestational age at delivery (*p* = 0.006) compared with homologous cycles. A higher frequency of placental hypoplasia was observed among heterologous pregnancies (55% vs. 14%; *p* = 0.03). A near-universal prevalence (95–100%) of MVM and fibrinoid deposition was observed across all ART placentas. **Discussion**: ART pregnancies, particularly those achieved through heterologous cycles, appear to represent a higher-risk category associated with accelerated placental aging and impaired placental growth. These findings highlight the importance of comprehensive counseling and informed consent, and suggest that routine histopathological placental examination may be valuable to improve neonatal surveillance and clinical management.

## 1. Introduction

Infertility is a significant global health issue, affecting approximately 8–12% of reproductive-aged couples worldwide, with psychological, social, and economic consequences for those affected [1]. The World Health Organization defines infertility as a disease of the male or female reproductive system, characterized by the failure to achieve pregnancy after 12 months or more of regular, unprotected sexual intercourse [2].

The causes of infertility in both men and women are numerous and heterogeneous [3].

The most important negative predictor of fertility is increasing maternal age at conception. However, lifestyle and environmental factors also play a significant role [3,4]. 

The primary goal of infertility treatment is to identify and, whenever possible, correct the underlying cause, in order to restore the couple’s natural ability to conceive.

However, this is not always feasible, and medically assisted procreation plays a central role in the management and treatment of infertile couples.

The American Centers for Disease Control and Prevention define Assisted Reproductive Technologies (ART) as fertility treatments involving the manipulation of oocytes or embryos [5]. These techniques are fundamental in the treatment of infertility and have revolutionized reproductive medicine, providing effective solutions for conditions such as tubal factor infertility, male factor infertility, endometriosis, ovulatory disorders, and unexplained infertility [6,7,8].

In vitro fertilization (IVF) is the most used ART technique [5].

Both homologous and heterologous ART are generally considered safe; however, they are associated with specific maternal, obstetric, and perinatal risks. These risks are largely influenced by maternal age, underlying infertility, comorbidities, and treatment characteristics. Women who conceive through ARTs are at higher risk of complications including stillbirth, pre-eclampsia, preterm birth, low birth weight, congenital malformations, and cesarean delivery compared with spontaneous conception [8]. However, it remains uncertain whether these increased risks are primarily attributable to ART procedures themselves or to the underlying infertility-related conditions affecting the couple. Even in large cohort studies, distinguishing the individual contributions of these factors remains challenging [8]. 

The placenta plays a fundamental role in pregnancy, acting as the interface between the mother and the fetus by enabling nutrient transfer, gas exchange, and the regulation of immune tolerance and endocrine function [9]. Consequently, placental examination provides crucial insights into the pathophysiology of pregnancy complications and represents a valuable tool for both clinical practice and research [10]. 

With the increasing global use of Assisted Reproductive Technologies, there has been growing interest in understanding how these interventions may affect placental development and function. 

In particular, ART procedures themselves may influence placental development. Factors such as ovarian stimulation, in vitro embryo culture conditions, cryopreservation, and timing of embryo transfer have been implicated in altering early placentation [11]. Therefore, systematic examination of placental histopathology can identify distinct lesions—such as vascular malperfusion, inflammatory changes, and abnormalities in trophoblast development—that may help differentiate alterations potentially associated with ART procedures from those attributable to underlying infertility or maternal conditions. These additional pathological findings may provide further insight into the biological mechanisms underlying the association between ART and adverse pregnancy outcomes, improve risk stratification, and support the development of more targeted monitoring and management approaches in ART pregnancies. Furthermore, the diversity of ART populations makes it difficult to identify consistent placental patterns, and it is still unclear whether specific placental changes can be reliably associated with particular ART exposures.

The primary aim of this study was to compare adverse maternal and fetal outcomes, as well as histopathological findings between spontaneous pregnancies and pregnancies conceived through ART, distinguishing between homologous and heterologous procedures.

## 2. Materials and Methods

### 2.1. Study Population

In this retrospective cohort study, we analyzed clinical and placental histopathology data from patients who underwent medically assisted reproduction techniques and delivered between November 2016 and August 2024, at the Department of Obstetrics and Gynecology, A. Gemelli Hospital, Rome, Italy.

Of the 97 cases initially identified, 13 twin pregnancies and 4 pregnancies ending in spontaneous abortion were excluded.

A total of 80 singleton ART pregnancies were therefore included in the final analysis.

In particular, pregnancies achieved through both homologous and heterologous assisted reproductive technologies (ARTs) were obtained using either In Vitro Fertilisation with Embryo Transfer (IVF-ET/FIVET) or Intracytoplasmic Sperm Injection (ICSI).

This group was compared with a retrospective cohort of 80 patients who conceived spontaneously, matched for maternal age and with complete clinical data, and who delivered during the same period and at the same institution. Only term pregnancies delivered at or beyond 37 completed weeks of gestation were included. 

In both groups were excluded cases with fetal malformations and cases with incomplete clinical data. 

The ART group was further divided into two subgroups:

- 45 patients treated with homologous ART;
- 35 patients treated with heterologous ART.

### 2.2. Clinical Data Collection and Evaluation

The following maternal and neonatal characteristics were collected and analyzed: maternal age, parity, pre-gestational maternal height, and weight (BMI), diagnosis at admission, pre-existing maternal conditions, and pregnancy outcomes (gestational age at delivery, mode and indication of delivery, birth weight, birth weight percentile, Apgar scores at 1 and 5 min, admission to neonatal intensive care [NICU]).

Gestational age (GA) was calculated based on the last menstrual period and confirmed by the first-trimester ultrasound.

Newborns with a birth weight below the 10th percentile (according to national growth curves for singleton pregnancies) were classified as small for gestational age (SGA).

### 2.3. Collection and Evaluation of Placental Histopathology 

Placentas were collected immediately after delivery and subjected to pathological examination. 

Macroscopic examination of the placental parenchyma was performed according to the Amsterdam Placental Workshop Group Consensus Statement [12]. Each sample was routinely evaluated using five hematoxylin and eosin–stained (H&E) slides. When macroscopic examination of the placenta revealed lesions, these areas were additionally sampled, and further slides containing the corresponding lesions were prepared for microscopic evaluation.

Placentas with a weight below the 10th percentile (based on placental weight curves by gestational age at delivery) were classified as showing placental hypoplasia [13]. 

Histological lesions were classified according to Benton et al. [14], as follows:

- category 1: evidence of maternal vascular malperfusion;
- category 2: evidence of maternal decidual arteriopathy;
- category 3: implantation site abnormalities;
- category 4: evidence of ascending intrauterine infections;
- category 5: evidence of placental villous maldevelopment;
- category 6: evidence of fetal vascular malperfusion;
- category 7: evidence of utero-placental separation;
- category 8: fibrinoid deposition;
- category 9: intervillous thrombi;
- category 10: evidence of chronic inflammation.

All histological slides were independently reviewed by two experienced pathologists (EN and VA), each blinded to the other’s assessments, in order to minimize observer bias and enhance diagnostic reliability. In cases of diagnostic uncertainty or discrepancy, a collegial review process was undertaken to establish the final diagnosis.

All observed lesions were then recorded in the final histopathological report.

## 3. Statistical Analysis

All clinical and histopathological data were compared between the spontaneous pregnancy group and the ART group.

Subsequently, the same variables were analyzed within the ART group by comparing patients who conceived through homologous medically assisted reproduction techniques with those who conceived through heterologous techniques. 

Continuous variables are presented as mean ± standard deviation, while categorical variables are reported as frequency (percentage). Categorical variables were compared using the Chi-square or Fisher’s exact test, as appropriate. Continuous variables were compared using Student’s *t*-Test. A *p*-value < 0.05 was considered statistically significant.

Statistical analysis was performed through JMP (v. 19, JMP Statistical Discovery LLC, Cary, NC, USA).

### 3.1. Clinical Data Results 

Maternal age, BMI, gestational age at delivery, neonatal birth weight and percentile, Apgar scores at 1 and 5 min, rate of small for gestational age (SGA) newborns, and NICU admission rate were overall comparable between the two groups.

None of the newborns in the control group required NICU admission, whereas 15% of the newborns in the ART group were admitted for observation.

A higher incidence of SGA newborns was also observed in the ART group, with an increase of 6% compared to the control group.

Additionally, the rate of cesarean sections was slightly higher among pregnancies achieved through medically assisted reproduction (+7%).

To further investigate these findings, the ART group was subdivided into two cohorts: patients who underwent homologous ART and those who underwent heterologous ART.

Statistical analysis of the clinical data for these two subgroups is presented in Table 1.

The mean maternal age was significantly higher in the heterologous ART group (42.6 years) compared with homologous ART group (37.8 years), (*p*-value of 0.007). Gestational age at delivery was also significantly lower in the heterologous ART group (37.05 weeks) compared to the homologous ART group (39.28 weeks) (*p*-value = 0.006).

BMI, as well as Apgar scores at 1 and 5 min, were comparable between the two groups. Similarly, neonatal birth weight and birth weight percentiles did not differ significantly; however, a decreasing shift was observed in pregnancies achieved through heterologous ART. Notably, among newborns admitted to the NICU for prematurity in the ART group (see Table 1), the majority were born from heterologous ART pregnancies (20% vs. 7%). Likewise, the incidence of small for gestational age (SGA) newborns was higher in the heterologous ART group compared with the homologous ART group (15% vs. 7%).

### 3.2. Placental Histology Results

Clinical data derived from the statistical analysis of the study populations were then correlated with placental histopathological findings, including both macroscopic and microscopic features.

Initially, macroscopic placental findings were evaluated. 

Statistical analysis comparing placentas from the homologous and heterologous ART groups showed similar placental weight and percentile values; however, lower values were observed in the heterologous ART group (490.75 g vs. 564.9 g; 18.25th vs. 26.8th percentile). The incidence of placental hypoplasia differed significantly between groups, being higher in the heterologous ART group (55%) compared with the homologous ART group (14%) (*p*-value = 0.03).

**Figure 1:**
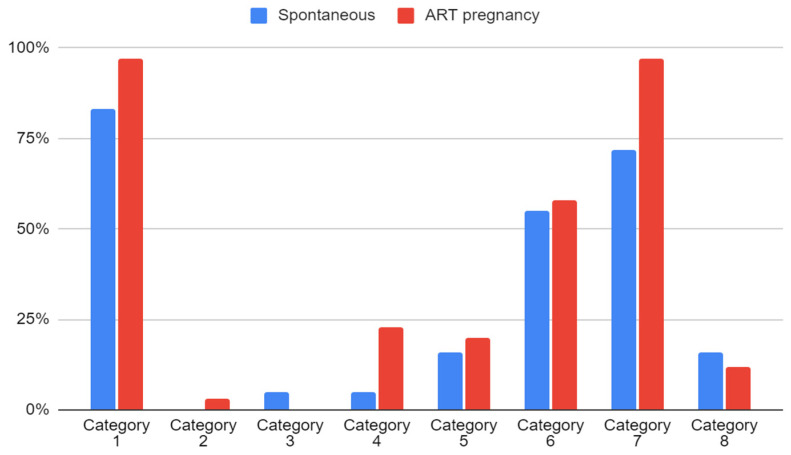
Different prevalence of placental lesions between spontaneous and ART pregnancies.

Following the assessment of macroscopic features, statistical evaluation was extended to the microscopic placental findings (Figure 1). The incidence of histological lesions corresponding to categories 1, 2, 4, 5, and 6 according to Benton et al. [14] was broadly similar between the two groups. However, an overall increase in these lesions was observed in placentas from ART pregnancies.

Lesions classified as category 1 (maternal vascular malperfusion; Table 2) and category 7 (evidence of utero-placental separation, such as retroplacental hematoma) were the most frequently observed findings in the ART group and were significantly more common compared with spontaneous pregnancy group (97% vs. 72%; *p*-value = 0.015).

**Figure 2:**
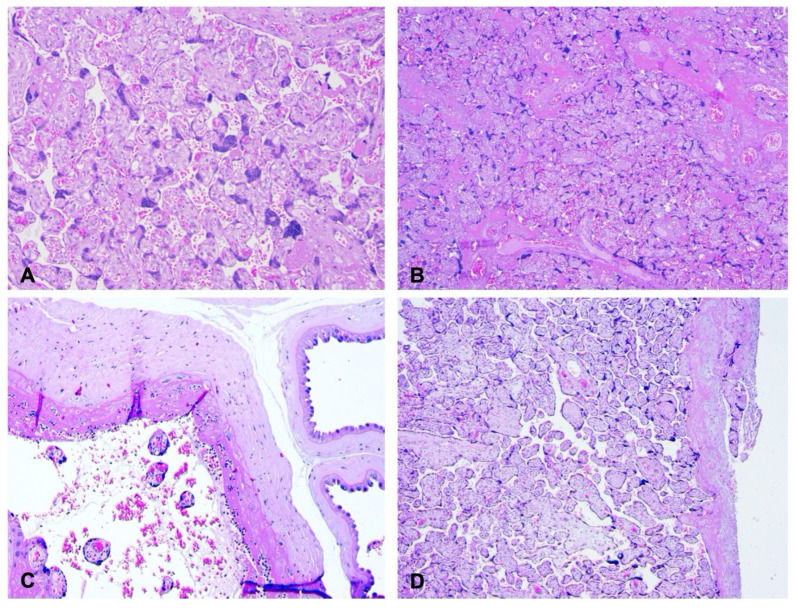
Histological images of ART-placentas; (**A**,**B**): agglutination and increased syncytial knots (lesions classified as category 1); (**C**): chorionitis and subchorionitis (lesions classified as category 4); (**D**): delayed villous maturation (GA 38 + 3 weeks; lesion classified as category 5) (H&E staining (**A**–**D**): magnification 10×).

Category 4 lesions (evidence of ascending intrauterine infection) were more frequently observed in ART pregnancies compared with spontaneous pregnancies (23% vs. 5%). Similarly, category 5 lesions (evidence of placental villous maldevelopment) were also more common in the ART group (20% vs. 16%) (Figure 2).

**Figure 3:**
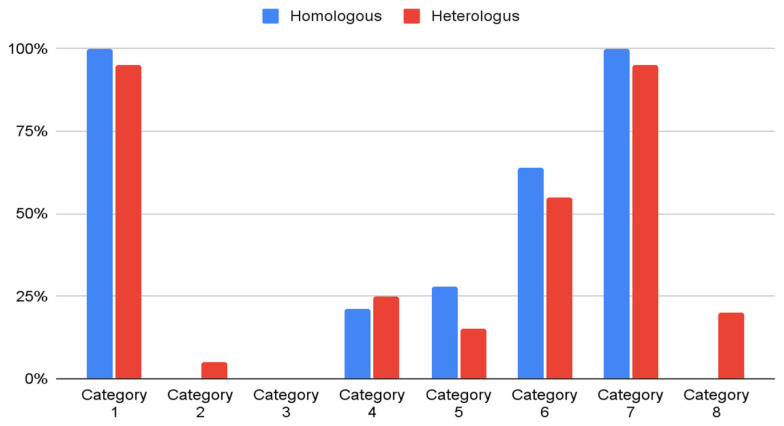
Different prevalence of placental lesions between homologous and heterologous ART pregnancies.

Further analysis comparing homologous and heterologous ART pregnancies (Figure 3) showed that the overall incidence of lesions across Benton et al. [15] categories was com-parable between the two subgroups.

Category 1 (maternal vascular malperfusion), category 5 (villous maldevelopment), category 6 (fetal vascular malperfusion), and category 7 (utero-placental separation) lesions were more frequently observed in the homologous ART group. In contrast, category 2 (maternal decidual arteriopathy) and category 8 (fibrinoid deposition) lesions were more commonly found in the heterologous ART group. 

No significant differences were observed in inflammatory or infectious lesions, suggesting that ART does not appear to be associated with an increased risk of placental inflammation.

## 4. Discussion

Our study demonstrated a significant association between assisted reproductive technologies (ART) and adverse pregnancy outcomes, particularly with respect to birth weight, gestational age at delivery, and placental histopathological findings.

Consistent with previous studies [15], pregnancies conceived through ART were associated with a higher rate of preterm birth (mean gestational age: 37.97 vs. 38.72 weeks) [16] and a lower birth weight than spontaneously conceived pregnancies [6,7,17]. Although the higher prevalence of small for gestational age (SGA) newborns in the ART group did not reach statistical significance, it suggests a tendency toward impaired fetal growth.

Although the following associations did not reach statistical significance, they may represent exploratory findings and are therefore reported for hypothesis-generating purposes. The higher rate of preterm birth in ART pregnancies was also reflected in a higher rate of neonatal intensive care unit (NICU) admission due to prematurity (15% vs. 0%), with an even higher proportion observed among heterologous ART pregnancies (20%). As regards the mode of delivery, the literature agrees that ART pregnancies are associated with higher rates of labor induction and C-section, with pathological CTGs frequently representing an indication for operative delivery [18,19,20]. On this point too, our data are consistent with these reports, as the incidence of C-section was higher in ART group than in the spontaneous conception one (67% vs. 60%).

The main finding of our study is that pregnancies achieved through ART should be considered high-risk pregnancies, given the increased incidence of NICU admission, pre-term birth, and SGA newborns. 

Regarding SGA fetuses, the recent literature suggests that in pregnancies conceived via fresh blastocyst transfer (BT), the proportion of SGA fetuses increases more markedly with advancing gestational age, whereas in frozen–thawed BT pregnancies the increase is more gradual and progressive [21]. In addition, pregnancies conceived after fresh blastocyst transfer may require enhanced fetal surveillance due to increased uterine artery pulsatility index and a higher risk of SGA [22].

These hypotheses can support the inclusion of placentas from ART pregnancies among the categories for which routine histopathological examination should be recommended.

Regarding the macroscopic characteristics of the placentas examined, our findings differ from those reported in the literature, particularly with respect to placental weight.

According to some authors [23,24], the use of assisted reproductive technologies is associated with increased placental weight and thickness, possibly as a consequence of the effects of hormonal stimulation during controlled ovarian hyperstimulation on endometrial development. In contrast, in the present study, placental weight was significantly lower in the ART group, and placental hypoplasia was more frequently diagnosed than in spontaneously conceived pregnancies. 

These findings may be clinically relevant, as placental size is widely recognized as an indicator of both placental function and fetal well-being [25]. This discrepancy between our results and those reported in previous studies may be explained by differences in study populations, particularly the higher mean maternal age in our cohort. In addition, advanced maternal age has been associated with lower placental weight; this data could suggest that, in ART pregnancies, the negative impact of maternal age on placental development may override the potentially growth-promoting effects of hormonal stimulation. Nevertheless, maternal age remains an important potential confounder, and this finding should therefore be interpreted as hypothesis-generating. In the absence of multivariable adjustment, it is not possible to determine the independent contribution of maternal age to the observed associations. Future studies incorporating multivariable analyses will be necessary to clarify its independent effect. Histopathological analysis revealed a higher incidence of lesions related to maternal vascular malperfusion (MVM) in the ART group. MVM results from defective spiral artery remodeling and impaired uteroplacental perfusion. In normal pregnancy, extravillous trophoblasts invade the decidua and remodel the spiral arteries into dilated, low-resistance vessels, ensuring adequate placental blood flow [15]. Inadequate remodeling leads to intermittent perfusion, ischemia–reperfusion injury, and increased oxidative stress [26].

Oxidative stress represents a key pathogenic mechanism linking abnormal placentation to the characteristic histopathological features of MVM, including syncytial knots formation, distal villous hypoplasia, and placental infarction [14,15]. These abnormalities may be related either to the underlying infertility or to ART procedures themselves. Specifically, factors such as embryo manipulation, in vitro culture conditions, and hormonal stimulation may disrupt early placental development and implantation [3].

Pregnancies achieved through ART occur within a distinct endocrine environment that may interfere with these physiological processes. In programmed frozen embryo transfer cycles, the absence of a corpus luteum results in the lack of corpus luteum–derived vasoactive mediators, such as relaxin and vascular endothelial growth factor, which play crucial roles in systemic and uterine vascular adaptation [26].

In addition, the increase in syncytial knots was the most observed lesion in ART pregnancies. A similar observation can be made regarding the different incidence of category 8 lesions (fibrinoid material deposits) between the two groups. These lesions were more frequently observed in ART pregnancies compared with spontaneous pregnancies (97% vs. 72%; *p*-value = 0.015). Moreover, according to the literature, such lesions may be associated with placental ischemia or chronic alteration in intervillous perfusion, as well as with conditions characterized by villous damage, since fibrinoid material deposits replace the damaged villi [27].

When analyzing ART placentas separately, both lesions related to maternal vascular malperfusion (Cat. 1) and those due to fibrinoid material deposition (Cat. 8) were present in 100% of homologous ART pregnancies and in 95% of heterologous ART pregnancies. Although a slight difference was observed between subgroups, both lesion categories were present in nearly all ART placentas.

The main finding of our study is that the placentas from pregnancies achieved through ART techniques are, on average, of lower weight, if not hypoplastic, and show lesions (Cat. 1 and Cat. 7) typically observed in term placentas and/or those affected by hypoxic problems. This occurs despite a lower mean gestational age at delivery compared with controls and in the absence of pathological conditions typically associated with hypoxic damage. Based on this evidence, we hypothesize that the coexistence of these two histopathological lesions is likely related to a state of placental hypoxia due toaltered placental vascularization, with the *primum movens* plausibly originating in the earliest stages of implantation [25].

A distinctive and novel aspect of this study is the detailed comparison between homologous and heterologous ART techniques. While existing literature frequently clusters all ART pregnancies into a single high-risk category, our data reveals that heterologous (oocyte donation) pregnancies exhibit a distinct and more severe clinico-pathological profile.

Our findings demonstrate that placental hypoplasia was significantly more frequent in the heterologous group (55%) compared to the homologous group (14%, *p* = 0.03). This observation may suggest that the use of donor oocytes could be associated with additional biological variables potentially influencing placental growth, beyond the stressors inherent to the ART process itself. This potential “donor related effect” may manifest as reduced placental volume and could be associated with the lower gestational age at delivery observed in the heterologous cohort (37.05 vs. 39.28 weeks). However, this association requires confirmation in adjusted analyses accounting for maternal age and other potential confounders in order to better understand their contribution.

The near-universal presence of Category 1 (MVM) and Category 8 (fibrinoid deposition) lesions in both groups (95–100%) could suggest that the “vascular hit” of ART occurs regardless of oocyte source. However, the significantly higher maternal age in the heterologous group (42.6 years) suggests a synergistic risk: the combination of an aged uterine environment and an allogeneic (donor) embryo may further impair spiral artery remodeling.

Therefore, we hypothesize that, while homologous ART placentas show morphological features consistent with a potentially accelerated placental aging process, heterologous ART placentas show a combined phenotype of accelerated aging and growth restriction. This distinction may be critical for clinical practice suggesting that heterologous pregnancies may require more intensive third-trimester surveillance and earlier consideration of delivery compared with their homologous counterparts.

From a medico-legal perspective, our findings, highlighting that ART pregnancies are high-risk, with an increased likelihood of cesarean delivery and a significantly higher risk of complications in heterologous cycles, underscore the critical importance of informed consent. In this context, informed consent transcends standard clinical protocols. For instance, within the European Union, ART procedures are predominantly performed in private facilities; consequently, consent is further governed by the Consumer Protection Directive, which mandates the provision of exhaustively detailed information to the patient [28,29].

## 5. Conclusions

This study aimed to evaluate the histological findings of placentas obtained through medically assisted reproduction.

In this retrospective cohort, pregnancies conceived through ART showed a higher frequency of selected adverse perinatal outcomes and placental histopathological lesions compared with spontaneous pregnancies. Among ART pregnancies, heterologous cycles were associated with older maternal age, earlier delivery, and a higher frequency of placental hypoplasia. Because of the retrospective design, small sample size, multiple comparisons, and lack of adjustment for confounding variables, these findings should be considered exploratory and require confirmation in larger prospective studies.

Further prospective studies are required to better understand the mechanisms underlying these associations and to identify potential strategies for improving maternal and neonatal outcomes in this population.

Given the rising prevalence of ART and its association with unique placental pathologies, systematic placental examination may be considered as a standard component of post-delivery evaluation in these pregnancies. This approach may improve immediate clinical management and contribute to research efforts aimed at optimizing ART techniques and improving maternal–fetal health outcomes.

This study underscores that pregnancies achieved through ART represent a distinct high-risk obstetric category. Our findings demonstrate a significant association between ART and adverse perinatal outcomes, specifically preterm birth, lower birth weight, and a higher incidence of NICU admissions.

Importantly, histopathological analysis revealed a near-universal prevalence of Maternal Vascular Malperfusion and fibrinoid deposition in ART placentas. These findings suggest that the combination of advanced maternal age and the lack of maternal–fetal genetic identity may further impair spiral artery remodeling.

From a clinical and medico-legal standpoint, these results have two major implications:

1. Systematic histopathological examination of the placenta may be considered as part of the clinical evaluation in ART deliveries, as it could contribute to better understand the “vascular hit” and help future neonatal surveillance.
2. Given the heightened risk profile—particularly in heterologous cycles—the in-formed consent process must be exceptionally rigorous and detailed.

Further prospective research is warranted to isolate the epigenetic and hormonal drivers of these placental alterations, ultimately aiming to optimize ART protocols and improve long-term maternal–fetal health.

## Figures and Tables

**Table 1:** Correlation of fetal outcomes and maternal characteristics in homologous and in heterologous ART pregnancies.

	Homologous	Heterologous	*p*-Value
Median maternal age	37.86	42.6	0.007 *
BMI	24.24	23.18	0.649
Gestational age at delivery	39.28	37.05	0.006 *
Newborn weight	3247.5	2841.75	0.61
Apgar 1′	8.86	8.47	0.092
Apgar 5′	9.71	9.37	0.09
C-section	57%	71%	0.273
SGA	7%	15%	0.627
NICU	7%	20%	0.379

Note: bold text and the asterisk (*) indicate statistically significant correlations (*p*-value < 0.05).

**Table 2:** Differences in lesions belonging to category 1 according to Benton between spontaneous and ART pregnancies.

Lesions (Category 1)	Odds Ratio (OR)	95% CI	*p*-Value (Fisher’s)
Infarcts	23.98	[1.38–416.64]	0.0014
Villous agglutination	0.31	[0.13–0.76]	0.0128
Distal villous hypoplasia	2.21	[0.84–5.81]	0.1589
Accelerated maturation	9.47	[0.50–178.88]	0.1203
Increased syncytial knots	0.66	[0.35–1.24]	

## Data Availability

The original contributions presented in this study are included in the article. Further inquiries can be directed to the corresponding author.

## Abbreviations

ARTs	Assisted Reproductive Techniques
SGA	Small for Gestational Age
NICU	Neonatal Intensive Care Unit
GA	Gestational Age
BMI	Body Mass Index
H&E	Hematoxylin and Eosin
MVM	maternal vascular malperfusion
